# Long-Term Outcome of Combined (Percutaneous Intramyocardial and Intracoronary) Application of Autologous Bone Marrow Mononuclear Cells Post Myocardial Infarction: The 5-Year MYSTAR Study

**DOI:** 10.1371/journal.pone.0164908

**Published:** 2016-10-20

**Authors:** Mariann Gyöngyösi, Georgiana-Aura Giurgea, Bonni Syeda, Silvia Charwat, Beatrice Marzluf, Julia Mascherbauer, Andras Jakab, Abelina Zimba, Márta Sárközy, Noemi Pavo, Heinz Sochor, Senta Graf, Irene Lang, Gerald Maurer, Jutta Bergler-Klein, on behalf of the MYSTAR investigators

**Affiliations:** 1 Department of Cardiology, Internal Medicine II, Medical University of Vienna, Vienna, Austria; 2 Department of Angiology, Internal Medicine II, Medical University of Vienna, Vienna, Austria; 3 Department of Biomedical Imaging and Image-guided Therapy, Medical University of Vienna, Vienna, Austria; 4 Metabolic Diseases and Cell Signaling Group, Department of Biochemistry, Faculty of Medicine, University of Szeged, Szeged, Hungary; Freeman Hospital, UNITED KINGDOM

## Abstract

**Objective:**

The long-term (5-year) outcome of early (3–6 weeks after acute myocardial infarction [AMI], BM-MNC Early group) and late (3–4 months after AMI, BM-MNC Late group) combined (percutaneous intramyocardial and intracoronary) delivery of autologous bone marrow mononuclear cells (BM-MNCs) was evaluated in patients with ejection fractions (EF) between 30–45% post-AMI.

**Methods:**

Major adverse cardiac and cerebrovascular events (MACCE) and hospitalization were recorded. Left (LV) and right (RV) ventricular function were measured by transthoracic echocardiography. Cardiac magnetic resonance imaging (MRI) and myocardial single photon emission computed tomography was performed in a subgroup of patients. Pre-cell therapy myocardial voltage values of treated areas (assessed by NOGA mapping) were correlated with clinical outcome.

**Results:**

Five-year MACCE incidences (7.4%. vs 24.1%) and the composite of all adverse events (11.1% vs 27.6%) were not different between the Early and Late treatment groups. The significant LV-EF increase at 1-year follow-up was preserved at the 5-year control (from baseline to 5-year: 5.3%, 95% CI:0.5–10.1, and 5.7%, 95% CI:1.7–9.6, p<0.05 in the Early and Late groups, respectively), with no significant changes between 1- and 5-year follow-ups. Similarly, RVEF increased significantly from baseline to the 5-year follow-up (Early group: 5.4%, 95% CI:1.0–9.6; and Late group: 8.4%, 95% CI:4.5–12.3). Lower baseline levels of myocardial viability of the treated cardiac area (6.3±2.4 vs 8.2±3.0 mV, p<0.05) were associated with incidence of MACCE.

**Conclusions:**

Percutaneous combined delivery of autologous BM-MNCs is feasible and safe after 5 years, and may result in sustained improvement of cardiac function at 5 years in patients with low EF post-AMI (Clinicaltrials.gov NCT01395212).

## Introduction

Recent meta-analyses of randomized clinical studies including patients with acute myocardial infarction (AMI) treated with/without intracoronary delivery of autologous bone marrow (BM) mononuclear cells (MNC) introduced debate about the efficacy of this cardiac regenerative treatment mode [1,2]. However, treatment of patients with chronic ischemic heart failure receiving either intracoronary or intramyocardial delivery of cells of diverse type (BM-MNC, autologous or allogeneic BM, or adipose tissue-derived mesenchymal stem cells) seems to be efficient in terms of significant increases in global left ventricular (LV) ejection fraction (EF) as shown by recently published meta-analyses [3,4]. Intramyocardial injection of regenerative cells has various advantages over intracoronary application such as negligible wash-out, with a higher retention rate of the cells in the myocardium [5,6]. However, intracoronary delivery is clinically more attractive because of its simplicity [7–9]. Our MYSTAR, prospective, single-blind study included patients with recent AMI randomized to receive combined (intramyocardial followed by intracoronary) autologous BM-MNCs injections early (3–6 weeks, BM-MNC Early group) or late (3–4 months BM-MNC Late group) post-AMI with the aim of exploiting the advantages of both delivery modes [5]. The initial study was completed with a 1-year follow-up in 2008 [5,10,11], demonstrating moderate but significant improvement in infarct size and LV function [5].

Most cardiac regeneration studies report short-term (3–6 month) follow-up results while some mid-term (over 1-year) follow-up studies suggested a possible loss of the initial benefits of cardiac cell-based treatment [12]. Up to now, only few trials monitored patients who received BM or peripheral blood-origin cell treatments for over 2 years. The BOOST (5 years), ASTAMI (3 years), MAGIC (2 years), Cao study (4 years), MAGIC Cell3-DES (5 years), REPAIR-AMI (2 years), TOPCARE-AMI (5 years), Plewka study (2 years) and the STIM studies reported heterogeneous long-term results (Table A in S1 File) [13–21].

Right ventricular (RV) dysfunction develops in approximately half of patients with MI in the absence of posterior or inferior wall ischemia [22,23] and is attributed to multifactorial reasons such as an increase in left atrial pressure and afterload, altered preload conditions, and RV myocardial stunning. Several studies identified RV dysfunction as a strong prognostic factor for in-hospital and mid- and long-term mortality, development of heart failure, and LV infarct burden [22,23]. Currently, no data are available about the changes in RV function after cell-therapy post AMI.

In this prospective study, we investigated the 5-year clinical outcome, LV and RV systolic and LV diastolic function, and infarct size in patients included in the MYSTAR study. This is the only trial that used the combined delivery route and investigated the long term effect of percutaneous intramyocardial and intracoronary delivery of BM-MNCs on LV and RV function.

## Methods

### Study design

**Fig 1:**
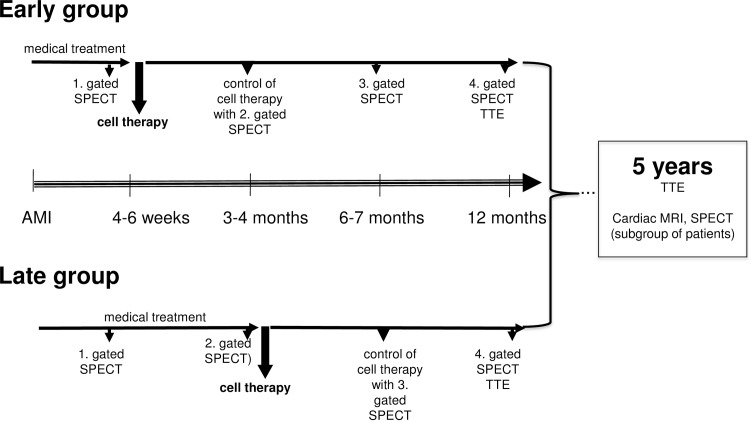
Study design.

This is a single-center, 5-year follow-up study (MYSTAR-5-YEAR, clinicaltrials.gov NCT01395212) including patients who participated in the MYSTAR trial at the Medical University of Vienna (clinicaltrials.gov NCT00384982) (Fig 1) [5].

### Patients

The MYSTAR study design, inclusion and exclusion criteria, primary and secondary endpoints, and 1-year results are published elsewhere [5,10]. Briefly, patients with a recent AMI were included and randomized to Early (n = 30; BM-MNC therapy between 3 and 6 weeks) or Late (n = 30; BM-MNC therapy between 3 and 4 months) groups receiving autologous BM-MNC using combined delivery (intramyocardial followed by intracoronary) therapy a mean ± standard deviation (SD) of 32 ± 12 or 93 ± 15 days post ST segment elevation myocardial infarction (STEMI), respectively.

The main inclusion criteria were age >18 years, global LV EF between 30% and 45%, new persistent local wall motion disturbance, no significant coronary artery lesion of more than 50% diameter stenosis, and written informed consent.

The main exclusion criteria included previous heart surgery or AMI at the same location, regional wall motion abnormality outside the index AMI area, and severe renal, lung, or liver disease. Patients presenting with severe valve disease, hemoglobin level below 9 mg%, New York Heart Association (NYHA) class IV, or ventricular thrombus were also excluded.

The detailed cell administration protocol was published previously. Briefly, after harvesting of the BM and selecting the MNCs, patients in the Early and Late groups received 200.3±68.7 x10^6^ and 194.8±60.4 x10^6^ BM-MNCs intramyocardially followed by 1.3±0.68 x10^9^ and 1.29±0.41 x10^9^ BM-MNCs intracoronary, respectively [5]. The locations of the percutaneous intramyocardial injections were guided by 3-dimensional NOGA endocardial mapping as described previously [6]. The intracoronary infusions were given into the reopened infarct-related artery at a slow injection rate [5].

All patients were invited to participate in the 5-year control investigations. The 5-year follow-up study protocol was approved by the Ethik-Kommission der Medizinischen Universität Wien (EK Nr: 291/2011) and written informed consent was obtained from all study participants. The study was conducted according to the Declaration of Helsinki.

The patients underwent a series of 5-year follow-up tests including interviews (detailed medical history and assessment of current medication and NYHA and Canadian Cardiovascular Society [CCS] classifications), physical assessment, routine clinical laboratory tests, electrocardiography, and transthoracic echocardiography. Cardiac magnetic resonance imaging (MRI) and 99m-Sestamibi single photon emission computed tomography (SPECT) stress-rest SPECT was performed in a subgroup of patients where applicable.

### Study endpoints

The primary endpoint of the current follow-up study was the occurrence of major adverse cardiac and cerebrovascular events (MACCE) including all-cause death, re-AMI, target vessel revascularization (TVR), and stroke during the 5-year follow-up. Additionally, the incidence of all adverse events (including MACCE events, hospitalization and implantation of automatic implantable cardioverter defibrillator (AICD) or pacemaker (PM) were listed.

The secondary endpoints were improvements in clinical symptoms expressed as CCS and NYHA scores, changes in global LV- and RV-EF measured by echocardiography, size of the infarction determined by stress-rest SPECT, LV and RV volumes, segmental wall motion, and infarct transmurality and cardiac output measured by cardiac MRI.

### Two-dimensional transthoracic echocardiography (TTE)

Patients underwent a conventional TTE (Vivid 7, General Electric Inc), with acquisition of the apical chamber view in accordance with the current guidelines [24]. The left and right atrial and LV and RV end-diastolic diameters were measured. LV- and RV-EF were calculated with the biplane Simpson’s method from the 4- and 2-chamber views. LV wall motion score index (WMSI) was calculated according to updated guidelines. Diastolic LV function was documented by measuring the peak E- and A-wave velocities and the isovolumetric relaxation time by pulsed Doppler imaging.

At the 5-year follow-up, additional tissue Doppler imaging was performed using the apical four-chamber view. The peak early diastolic (e′) velocities were measured in the lateral and septal mitral annulus by calculating the mean values. Tricuspid annular plane systolic excursions (TAPSE) of the baseline, 1-year, and 5-year follow-up images were also measured from the 4-chamber view using M-mode, with the position of the cursor on the lateral tricuspid annulus. Structural abnormalities, such as myocardial calcification, were searched [25].

### Gated 99m-Technetium Sestamibi SPECT

A 2-day stress/rest (500–700 MBq 99mTc-Sestamibi) SPECT protocol was performed using dipyridamole infusion over 4–6 min (0.57 mg/kg/min by infusion pump) for the pharmacological stress study. The stress tests at inclusion and at follow-ups were performed with identical tracers, exercise loads, and cumulative dipyridamole doses. Infarct size was measured by quantifying the extent of tracer defect.

### Cardiac MRI

The MRI studies were performed on a 1.5-T, cardiac-dedicated, clinical magnetic resonance system (Sonata/Avanto, Siemens Medical Solutions, Erlangen, Germany). For the functional studies, 3 standard long-axis slices and a stack of contiguous short-axis slices (slice thickness: 10 mm, no gap, 30 phases/RR-interval) were acquired with electrocardiography-gated, steady-state, free-precession cine-images (repetition time 2.9 ms, echo time 1.2 ms, flip angle 80°, matrix 256×146, field of view typically 340 mm, band width 930 Hz/pixel) using a breath-hold technique. The late gadolinium enhancement (LE) images covering the LV were acquired 10 min after intravenous injection of 0.2 mM/kg gadolinium-diethyltriamine-penta-acetic acid (Magnevist, Bayer Schering Pharma, Germany) with a segmented inversion recovery sequence and inversion time optimized to null normal myocardial signal (TI between 200 ms and 300 ms). The LE images were acquired at end-systole and in the same position as the functional studies. For quantification of LV and RV function and volumes, the endocardial and epicardial contours were traced manually in end-systole and -diastole with dedicated software. The LV mass was calculated from the total myocardial volume multiplied by the specific gravity of the myocardium (1.05 g/ml). LE was defined as myocardial areas with signal intensity above the average of apparently normal myocardium plus 2 standard deviations using a 17-segment model, and expressed as a percentage of LV. The segmental analysis was carried out using Medviso Segment software in which the AHA 17-segment model was visually aligned and registered to the segmented LV myocardium. The segmental contraction velocity of the LV myocardium at the time point of peak ejection (cm/s) and the segmental infarct transmurality (line-based approach %) was calculated.

### Analyses of the NOGA® endocardial mapping and NOGA-guided myocardial SPECT

The myocardial viability expressed as unipolar voltage values (UPV), segmental wall motion (local linear shortening [LLS]) expressed as percentage, and infarct transmurality expressed as bipolar voltage values (BiP) were determined by NOGA endocardial mapping of the injected area [11,26].

### Statistics

All analyses were performed by an independent observer blinded to the randomization groups and time of the investigations. Continuous parameters are expressed as mean±standard deviation (SD); categorical variables are expressed as percentages. Descriptive statistics were used to describe the primary and secondary endpoints. The incidences of MACCE and all adverse events and continuous parameters were compared between the Early and Late groups; these were analyzed by chi-square and unpaired T-test, respectively. Baseline and follow-up data within the group were compared using ANOVA with repeated measurements supplemented by paired t-test. To analyze the effects of potential prognostic factors on the changes in the infarct size and in global EF at the 5-year follow-up (dependent variables) after excluding possible confounding factors, multivariate linear regression analysis was performed including the following independent variables: age, number of intra-myocardial and intra-coronary injected BM-MNCs, baseline values of UPV, LV EDV, and EF. Diabetes mellitus, male gender, randomization group (early or late) was correlated with MACCE using nominal logistic regression analysis.

For all tests, two-sided analyses were used and the significance level was set at *p* <0.05. The statistical analysis was carried out with SPSS® software for the Mac OS 10 operating system.

## Results

### Patients

Baseline characteristics for the entire patient population have been published previously [5,11]. The mean age of the patients at the 5-year follow-up was 57.5±9.1 years.

Four patients refused the follow-up investigations (all were symptom-free); therefore, the 5-year clinical follow-up was available for 56 (93.3%) patients. Five patients died during the 5-year follow-up; thus 51 patients completed the LV functional study.

### Primary Endpoint Results

The frequency of adverse events is listed in Table 1.

**Table 1:** Primary end point: occurrence of adverse events during the 5-year follow-up. In patients with multiple events during the 5-year FUP, the most serious major adverse event was entered as MACCE and the composite of all adverse events (one/patient) for each patient.

Adverse events during 5-year follow-up	Patients with 5-year follow-up (n = 56)	Patients with 5-year follow-up Early group (n = 27)	Patients with 5-year follow-up Late group (n = 29)
Adverse events			
PM/AICD implantation	5 (8.9%)	2 (7.4%)	3 (10.3%)
Hospitalization due to angina pectoris or heart failure	9 (16.1%)	3 (11.1%)	6 (20.7%)
**MACCE events** *(one/patient)*			
**TVR**	4 (7.1%)	1 (3.7%)	3 (10.3%)
**Re-AMI**	0 (0%)	0	0
**Stroke**	0 (0%)	0	0
**All cause death**	5 (8.9%)	1 (3.7%)	4 (13.8%)
***Composite MACCE***	**9 (16.1%)**	**2 (7.4%)**	**7 (24.1%)**
***Composite of all adverse events (one/patient)***	**12 (21.4%)**	**3 (11.1%)**	**8 (27.6%)**

PM: pacemaker. AICD: automatic implantable cardioverter defibrillator, TVR: target vessel revascularization; AMI: acute myocardial infarction; MACCE: major adverse cardiac and cerebrovascular events.

One patient received PM due to tachycardia-bradycardia syndrome. Four of the 56 patients participating in the 5-year investigation (7.1%) received AICD; 2 with progressive heart failure and malignant arrhythmias and 2 with low EF (baseline ≤35%) and ischemic cardiomyopathy for primary prevention of sudden cardiac death.

MACCE occurred in 9 of 56 patients (16.1%). Overall, 12 (21.4%) patients experienced adverse events. Some patients experienced multiple events such as implantation of AICD and TVR; in such cases, the most serious event was graded for the adverse event. The incidence of MACCE and the combined adverse events was not different between the Early and Late groups.

### Secondary Endpoint Results

The increase in global LV-EF and decrease in infarct size one year after cell therapy was preserved during the 5-year follow-up. In the entire group, the mean increase in LV-EF between pre-BM-MNC therapy and the 5-year follow-up was 5.4% (95% CI 2.3, 8.5; *p*<0.05) while this value between 1-year and 5-year follow-up was 1.9% (95% CI 1.4, 5.3; *p* = non-significant). The mean increase in LV-EF did not differ between the Early and Late groups, with both groups showing perceived improvement between pre-cell therapy and the 5-year follow-up (5.3%, 95% CI: 0.5, 10.1; *p*<0.05 in the Early group and 5.7%, 95% CI: 1.7, 9.6; *p*<0.05 in the Late group), while no changes between 1- and 5-year follow-up were seen in either group. However, due to lack of placebo control group, the observed LV and RV functional improvement can not be completely attributed to the cell therapy.

Similarly, the WMSI improved from baseline to the 1-year follow-up, and was preserved at the 5-year follow-up (Table 2) in both groups.

**Table 2:** Secondary end points of patients with LV functional studies (n = 51) 5 years after cardiac bone-marrow mononuclear cell (BM-MNC) therapy.

	Before BM-MNC therapyAll patients (n = 51) (Early vs Late group)	1 year post BM-MNC therapy All patients (n = 51) (Early vs Late group)	5 years follow-up All patients (n = 51) (Early vs Late group)
***Transthoracic echocardiography***			
LA diameter (mm)	53.6±8.9	48.3±7.8	47.1±7.7
	52.2±9.2 vs 55.0±8.4	47.6±8.2 vs 49.0±7.6	45.3±10.0 vs 48.9±4.5
LV EDD (mm)	54.3±7.0	51.9±7.3	53.1±8.6
	54.5±5.7 vs 54.0±8.2	51.2±52.6 vs 52.6±7.4	51.8±7.3 vs 54.0±9.4
RA diameter (mm)	51.9±6.3	54.0±6.9	53.7±6.4
	51.3±7.3 vs 52.6±5.1	53.2±6.7 vs 54.6±7.2	52.6±5.6 vs 54.5±6.9
RV EDD (mm)	32.7±5.1	32.6±3.7	32.7±4.5
	32.0±4.8 vs 33.4±5.3	32.1±5.4 vs 32.9±4.8	33.7±2.8 vs 32.2±5.2
LV EF (%)	39.2±9.3	41.5±8.3 ^a^	44.8±10.3 ^b^
	39.7±8.8 vs 38.7±7.7	42.7±10.5 vs 42.1±7.4	45.0±9.3 vs 44.3±6.9
LV WMSI	1.82±0.46	1.68±0.43	1.53±0.73 ^b^
	1.81±0.57 vs 1.84±0.39	1.65±0.40 vs 1.70±0.46	1.59±0.74 vs 1.50±0.73
RV EF (%)	42.3±7.1	45.5±8.3 ^a^	49.1±8.3 ^b^
	43.1±6.9 vs 41.4±7.2	46.5±9.2 vs 44.5±7.3	48.5±9.4 vs 49.8±7.2
Peak E velocity (m/s)	0.74±0.20	0.80±0.21	0.77±0.21
	0.75±0.22 vs 0.73±0.18	0.79±0.12 vs 0.81±0.2	0.76±0.22 vs 0.78±0.2
Peak A velocity (m/s)	0.83±0.15	0.80±0.15	0.81±0.13
	0.80±0.14 vs 0.86±0.16	0.77±0.12 vs 0.84±0.16	0.80±0.12 vs 0.82±0.14
E/A ratio	0.93±0.32	1.0±0.35	0.98±0.32
	0.97±0.15 vs 0.88±0.28	1.07±0.37 vs 1.0±0.33	0.99±0.35 vs 0.97±0.29
Isovolumetric relaxation time (ms)	99±9	96±8	100±10
	98±9 vs 99±10	94±8 vs 98±7	99±10 vs 102±11
E/e`ratio	not measured	not measured	13.4±4.9
			12.2±4.2 vs 14.5±5.3
TAPSE (cm)	13.2±4.2	15.2±4.2	15.6±4.1
	13.5±2.4 vs 12–7±4.8	15.5±6.4 vs 14.5±3.8	15.5±6.4 vs 15.0±3.8
***Clinical symptoms***			
NYHA	2.0±1.0	1.4±0.7	1.3±0.6 ^b^
	2.0±1.0 vs 2.1±0.9	1.5±0.7 vs 1.3±0.7	1.4±0.6 vs 1.3±0.6
CCS	1.2±0.7	1.2±0.5	0.9±1.0
	0.7±0.9 vs 1.7±0.9	1.2±0.6 vs 1.2±0.5	1.0±1.0 vs 0.8±0.9
Pro-BNP (pg/ml)	1323±1249	956±1057	465±537 ^b^
	1762±1732 vs 1031±742	1220±1439 vs 786±732	311±212 vs 578±670

^a^p<0.05: before BM-MNC therapy vs. 1 year post BM-MNC treatment

^b^p<0.05: before BM-MNC therapy vs. 5 years follow-up.

LA: diameter of left atrium; RA: diameter of the right atrium; LV: left ventricular; RV: right ventricular; EDD: end-diastolic diameter; EF: ejection fraction; WMSI: wall motion score index; TAPSE: tricuspid annular plane systolic excursion; NYHA: New York Heart Association of Heart Failure; CCS: Canadian Cardiovascular Society grading of angina pectoris.

**Fig 2:**
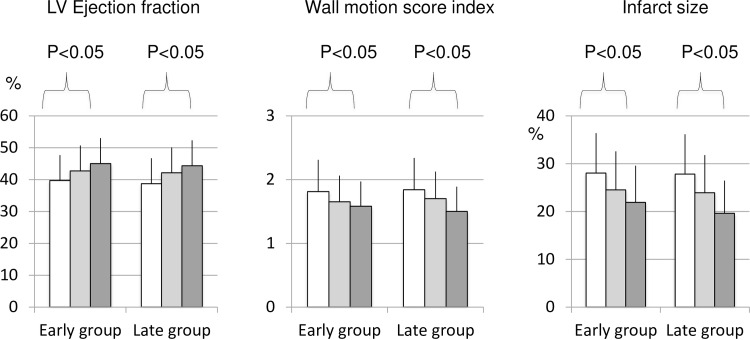
Left ventricular function and infarct size of patients randomized to Early or Late cell-therapy during the 5-year follow-up. **Patients were treated with combined delivery of autologous BM-MNC.** Global left ventricular (LV) ejection fraction, wall motion score index (measured by echocardiography), and infarct size (by single photon emission computed tomography) of patients in the Early and Late groups before receiving cardiac BMMNCs therapy and 1 and 5 years post cell therapy. No differences between the Early and Late group were observed.

The changes in LV function and infarct size pre-cell therapy, at 1 and 5-years FUP are displayed in Fig 2.

Trends towards decrease in LA and LV end-diastolic diameter were recorded. The mean increase in RV-EF in all patients was 3.2% (95% CI 2.0, 4.4; *p*<0.05) between baseline and the 1-year follow-up and 6.2% (95% CI 4.6, 7.8; *p*<0.05) between baseline and the 5-year follow-up, with no difference between the Early and Late groups (mean increase in EF from baseline to 5-year follow-up was 5.4%, 95% CI 1.0, 9.6; and 8.4%, 95% CI 4.5, 12.3, respectively).

A trend towards an increase in E/A (*p*<0.1) was measured at the 1-year follow-up with no further change during the next follow-ups regarding all diastolic function parameters. The peak early diastolic velocity (e`) was 6.1±1.5 cm/s resulting in an E/e`ratio of 13.4±4.9. No difference between groups was detected at the 5-year follow-up.

Echocardiography did not show any myocardial calcification or tumor formation attributable to cardiac cell-based therapy [27].

In association with a decrease in the N-terminal pro-BNP level, NYHA classification improved significantly at the 5-year follow-up, while the Canadian Cardiovascular Society classification did not change (Table 2), with no difference between the groups.

### Subgroup analyses

SPECT imaging showed a mean decrease in infarct size of 6.9% (95% CI −2.2, −11.7, p<0.01) between pre-BM-MNC therapy and the 5-year follow-up, and 3.8% (95% CI −9.0, 1.3; p = non-significant) between the 1- and 5-year post-BM-MNC therapy (Table 3), with no difference between the Early and Late groups.

**Table 3:** Left and right ventricular function in subgroup of patients. underwent single photon emission computed tomography (SPECT) and cardiac magnetic resonance imaging (cMRI) 5 years after cardiac bone-marrow mononuclear cell (BM-MNC) therapy.

	Before BM-MNC therapy	1-year post BM-MNC therapy	5-year follow-up
*SPECT (n = 31)*			
Infarct size (%)	27.4±10.7	24.3±11.6	20.1±11.8 ^b^
*cMRI (n = 26)*			
LV EDV (ml)	209±52.3	192.1±41.3	188.3±44.9
LV ESV (ml)	123.8±38.6	105.9±32.5	101.9±36.1
LV SV (ml)	85.2±19.9	86.1±15.4	86.3±16.2
LV EF (%)	41.4±6.5	45.7±7.2 ^a^	47.0±8.1 ^b^
Late enhancement (%)	25.0±16.9	20.9±14.4	18.0±12.6
LV CO (L/min)	5.0±1.3	5.2±1.2	5.6±1.2 ^b^
LV mass (g)	204±57	196±44	183±50
Infarct mass (g)	49±40	42±28	33±326 ^b^
RV EDV (ml)	159.2±32.0	150.3±19.7	146.9±21.3
RV ESV (ml)	87.7±34.7	72.5±18.1	66.4±12.6
RV SV (ml)	71.5±12.3	77.8±11.0	80.4±14.9
RV EF (%)	46.4±5.0	52.3±7.4 ^a^	54.8±6.0 ^b^
RV CO (L/min)	4.2±0.7	4.6±0.9	5.4±1.1 ^b^

^a^ p<0.05: before BM-MNC therapy vs. 1 year post BM-MNC treatment

^b^ p<0.05: before BM-MNC therapy vs. 5 years follow-up.

LV: left ventricular; RV: right ventricular; EDV: end-diastolic volume. ESV: end-systolic volume. SV: stroke volume; EF: ejection fraction. CO: cardiac output.

**Fig 3:**
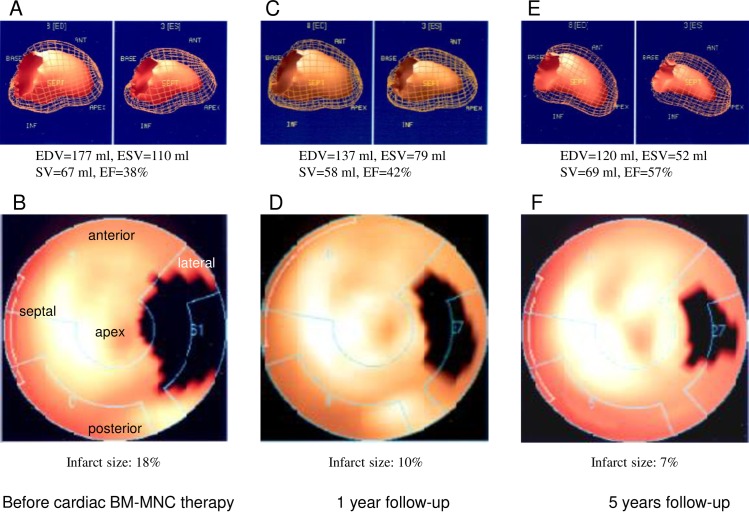
Gated ^99^m-Sestamibi myocardial scintigraphy of a patient with cardiac bone marrow mononuclear cell (BM-MNC) treatment. (A) and (B) Before combined cardiac delivery of autologous BM-MNC treatment; 3-dimensional calculation of LV volume (A) and polar map of infarct size (B). (C) and (D) At the 1-year follow-up, 3-dimensional calculation of LV volume (C) and polar map for infarct size (D). (E) and (F) At the 5-year follow-up, 3-dimensional calculation of LV volume (E) and polar map for infarct size (F).

Fig 3 displays the serial gated scintigraphy pictures of a patient treated with combined delivery of autologous BM-MNCs.

No stress-induced myocardial ischemia was detected during the 5-year investigations.

Similar to echocardiography, cardiac MRI showed a preserved improvement in LV-EF at 5 years when compared to baseline values, with a trend towards a decrease in LV end-diastolic and end-systolic volumes and increase in LV cardiac output (Table 3). RV-EF increased significantly 1-year post BM-MNC therapy, with preserved improvement during the 5-year follow-up. Since echocardiography did not show a difference between the Early and Late groups regarding LV and RV function at the 5-year follow-up, the groups were not compared in the subpopulation of patients available for myocardial perfusion scintigraphy or cardiac MRI.

**Fig 4:**
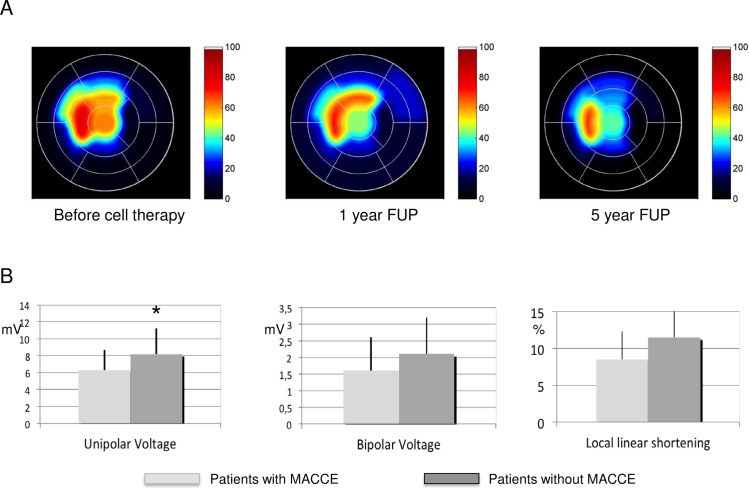
Schematic display of the segmental transmurality and NOGA electromechanical mapping-derived parameter. (A) Schematic display of the segmental transmurality using MathLab software, showing a decrease in infarct transmurality over 5 years in patients with acute myocardial infarction who were treated with combined delivery of cells. Summed data from 26 patients. (B) NOGA electromechanical mapping-derived parameter in patients with or without major adverse events during the 5-year follow-up. *p<0.05.

MRI with late enhancement revealed a significant decrease in infarct mass (Table 3), with a significant increase in segmental contractility (Table B in S1 File) and decrease in segmental infarct transmurality (Table 4) in 3 of the 17 myocardial segments (anteroseptal, anteroapical, and apex) (Fig 4).

**Table 4:** Segmental infarct transmurality determined by cardiac magnetic resonance imaging (n = 26).

Segment location	Before BM-MNC therapy	1 year post BM-MNC therapy	5 years follow-up
Basal anterior [%]	4.6±13.4	10.9±13.7	0.0±0.0
Basal anteroseptal [%]	4.6±12.0	11.1±13.1	0.4±1.4
Basal inferoseptal [%]	2.4±6.0	9.5±10.9	0.8±2.5
Basal inferior [%]	3.0±7.4	10.2±11.3	0.0±0.0
Basal inferolateral [%]	3.4±8.7	10.8±11.8	0.0±0.0
Basal anterolateral [%]	5.8±14.0	12.0±13.6	0.0±0.0
Mid anterior [%]	30.2±30.8	25.4±23.4	26.5±7.8
Mid anteroseptal [%]	46.2±32.7	37.4±28.8	31.4±26.7
Mid inferoseptal [%]	17.6±16.1	18.6±12.9	15.8±26.6
Mid inferior [%]	3.8±9.0	10.2±11.1	0.2±0.7
Mid inferolateral [%]	4.0±9.7	10.0±11.1	0.0±0.0
Mid anterolateral [%]	12.3±27.3	15.0±22.1	10.0±0.0
Apical anterior [%]	60.6±28.9	66.9±27.0	32.1±17.2 ^a^
Apical septal [%]	81.8±13.4	72.8±11.8	69.2±12.9 ^a^
Apical inferior [%]	23.4±16.4	21.3±13.3	26.4±21.0
Apical lateral [%]	6.5±15.1	10.5±13.1	0.9±2.1
Apex [%]	61.8±23.5	44.5±30.3	41.5±20.2 ^a^

^a^ p<0.05 betwwen baseline (before BM-MNC therapy) and 5-year follow-up values.

Multivariate linear regression analysis did not reveal prognostic factors of improvement in infarct size (measured in a subgroup of patients) or global EF. In contrast with the 3-month follow-up results, no correlation could be found between the number of injected BM-MNC and changes in infarct size in this subgroup of patients after 5 years.

Nominal logistic regression analysis could not identify predictive factors for occurrence of MACCE, all adverse events, or mortality. However, patients with 5-year MACCE in both groups had significantly lower baseline UPV values in the intramyocardially injected areas (6.3±2.4 vs 8.2±3.0 mV, *p*<0.05) (Fig 4).

## Discussion

This is the first prospective 5-year follow-up study of cell-based cardiac therapy delivered by percutaneous intramyocardial BM-MNC delivery followed by intracoronary cell injections. Our study demonstrated preserved global LV-EF, local wall motion abnormality, infarct size, and RV-EF. Trends towards decreased LV end-diastolic and end-systolic volumes, and LV end-diastolic and LA diameter were also shown, suggesting reverse remodeling post-AMI. To the best of our knowledge, this is the first study highlighting improved RV function and the long-term prognostic importance of the NOGA-derived parameter as higher baseline UPV was associated with event-free survival during the 5-year follow-up.

### Comparison of the follow-up results with other cardiac cell-based studies

Table A in S1 File summarizes the main and controversial results of published long-term follow-up of percutaneous cardiac cell-based treatment studies [13–21]. According to the obvious differencies between the trials regarding the applied cell types and delivery modes, duration of follow-up, and timing of treatment post-AMI, a direct comparison between studies is not feasible. To note, no long-term follow-up results are currently available in a randomized intramyocardial injection study that also included a placebo arm with which we could compare our study. Therefore, a direct association between intra-myocardial (or combined) cell therapy and persistent improvement in LV and RV function cannot be definitively concluded. Additionally, the strong and consequent clinical control of patients with guidelines-driven medicinal therapy, as well as revascularization might also have contributed to the supposed preserved benefit of the cell therapy and must be kept in mind. Unfortunately, not all patients could be controlled at the long-term follow-up, and five patients who initially had worse LV and RV function died; these facts might also influence the final results.

However, considering that the first 3-month follow-up in the Early group showed significantly better LV function when compared with the Late group, who were still on medical treatment 3 months post randomization, and the improvement in LV function and infarct size in the Late group after cell-based therapy, we could assume at least an indirect association between clinical improvement in patients treated with combined delivery of autologous BM-MNCs [5].

The results of a very recent meta-analysis that included trials with intracoronary delivery of BM-MNCs or mixed cell types raised questions about the efficacy of this therapy in patients with recent STEMI [1,2]. Our patient collective and study design differs substantially from those trials as the MYSTAR study included the earliest patients at 3 weeks (Early group) and up to 4 months (Late group) post-AMI and used combined cell delivery. Previously, we supposed that the intramyocardial cell delivery was responsible for the observed benefit [11].

Unfortunately, not all patients could be controlled at the long-term follow-up, and five patients who initially had worse LV and RV function died; these facts might also influence the final results.

As the AICD implantation frequency for primary prevention in ischemic cardiomyopathy ranges between 17–31%, and the incidence of malignant arrhythmias was low in our patient population, it is unlikely that the intramyocardial injections of BM-MNC were directly related to malignant cardiac arrhythmias [28]. Considering, that a 37-month mortality rate of 25.6% was reported in patients with LV EF between 36–45%, the total 8.3% mortality rate of our patients with baseline EF between 30–45% post-AMI might be considered as low, and the study safe [29].

We measured LV and RV function by transthoracic echocardiography as this functional investigation was available for all patients. Due to AICD or other implants, cardiac MRI could only be performed in a smaller group of patients (n = 26), while 31 patients underwent stress-rest SPECT. Recently, it was pointed out that the negative long-term outcome studies (ASTAMI, BOOST) used cardiac MRI to assess cardiac function [13,14]. In contrast, the positive outcome studies measured LV function with quantitative left ventriculography or echocardiography, methods that carry the bias of missing explicit objectivity. We also used transthoracic echocardiography; however, the LV-EF and its changes measured by MRI in a subgroup of patients were similar to that of the echocardiography measurements in the entire controlled patient cohort, even though patients with AICD due to low LV-EF were excluded from the MRI investigations.

To our knowledge, this is the first study demonstrating the improvement of RV function in patients with AMI and treated with cardiac cell-based therapy. The underlying mechanism might be the decreased afterload as a consequence of restoration of LV function, narrowing the gap between LV and RV cardiac output. Additionally, the improvement in the infarct size and local wall motion of the septal part of the anterior (96% of patients in the MRI subgroup) or inferior infarction might influence RV function.

A trend towards minor improvements in diastolic dysfunction was recorded 1-year post cell therapy; however, a clinically relevant robust change in diastolic function was not expected in patients with an initial LV-EF between 30% and 45%. However, our data are similar to those of the published diastolic dysfunction in other cell-therapy trials [14].

The decrease in heart failure parameters such as NT-proBNP or NYHA classification is an indicator of improvement in the patients’ clinical status. This might be due to treatment with medication to treat heart failure, with a consequent decrease in WMSC and increase in LVEF. According to the study design, all patients were treated with BM-MNCs; thus, we cannot determine whether the decrease in heart failure parameters can be attributed to the cell therapy.

### Subgroup analyses

Cardiac MRI in a subgroup of patients showed a significant reduction in the infarct mass from baseline to the 5-year follow-up, with a trend towards decreases in total LV mass, probably due to decreases in LV EDV, suggesting reverse remodeling. Parallel to decreases in infarct transmurality, the segmental contractility improved in 3/17 myocardial segments, supporting the echocardiographic data of the entire patient population. A trend towards decreased infarct size measured by myocardial scintigraphy and decreased infarct mass by cardiac MRI was observed at the 1-year follow-up. The LV EF decreased significantly at both the 1- and 5-year follow-up when measured by both echocardiography and MRI. Since the EF not only depends on the infarct size and mass, but also the geometry of the LV and adverse remodeling, the resting myocardial function expressed as EF is not necessarily directly associated with the morphological size of the infarction.

Subanalysis revealed significantly lower baseline UPV values of the intramyocardially injected area in patients experiencing MACCE. Because UPV is an index of myocardial viability, this finding is in accordance with previous statements on the predictability of myocardial viability on mortality measured by SPECT [26,30].

Higher numbers of cells (>10^8^) delivered intracoronary were reported to be in association with better long-term outcomes [31]. Higher numbers of intramyocardially injected cells predicted significant improvement in the infarct size at the 3-month follow-up [5]. Obviously, the initial impact of the cell number on cardiac function disappeared during the long-term follow-up. Of note, the infarct size determined by SPECT could be measured in a subgroup of the patients (n = 31) at the 5-year follow-up.

### Limitations

Patients in the MYSTAR study were randomized to Early and Late groups based on the principle that patients in the Late group were still on pharmacological treatment when the patients in the Early group were controlled 3-month post cardiac BM-MNC treatment. However, patients in the Late group were also treated 93±15 days post-AMI. The lack of a placebo group is a major limitation of our study; however, intramyocardial injection with a placebo substance raised ethical considerations in patients with recent AMI. Arbitrary selection of a control group from the 785 patients screened between 2002 and 2006 might have resulted in a severe bias in the study interpretation.

Although the number of our BM-MNC-treated patients is similar to that of the cell-treated arm of the randomized ASTAMI and TOPCARE-AMI, our study was underpowered regarding adverse events. However, according to a recently published statistical power calculation of myocardial regenerative studies, over 5000 participants should be included in each randomized group in order to detect any significant changes in mortality rates due to the reported minimal long-term incidence of death in patients treated with cardiac BM-MNCs [31].

## Conclusions

Percutaneous combined (intramyocardial and intracoronary) administration of autologous BM-MNCs is feasible and safe after 5 years, and may result in sustained improvement of cardiac function at 5 years in patients with low EF post-AMI. Higher initial voltage values in the cell injection area were associated with a lower long-term adverse event rate, which highlights the prognostic value of the myocardial viability factor derived from the electroanatomical mapping.

## Supporting Information

S1 File Table A in S1 File. Summary of long-term follow-up studies of patients treated with percutaneous cell therapy after acute myocardial infarction. Table B in S1 File. Segmental contraction velocity determined by cardiac magnetic resonance imaging (n = 26).
(DOCX)
