# Supplementary material for: Long-Term Outcome of Combined (Percutaneous Intramyocardial and Intracoronary) Application of Autologous Bone Marrow Mononuclear Cells Post Myocardial Infarction: The 5-Year MYSTAR Study
Source: PLoS One. 2016 Oct 20;11(10):e0164908. doi: 10.1371/journal.pone.0164908 (PMC5072601; doi:10.1371/journal.pone.0164908)
Supplement: S1 File — Table A in S1 File. Summary of long-term follow-up studies of patients treated with percutaneous cell therapy after acute myocardial infarction. Table B in S1 File. Segmental contraction velocity determined by cardiac magnetic resonance imaging (n = 26). (DOCX) [file pone.0164908.s001.docx]

**Table A. Summary of long-term follow-up studies of patients treated with percutaneous cell therapy after acute myocardial infarction**

| **Study** | **Injected cell type** | **FUP (year)** | **Delivery mode** | **Nr of FUP study pts** | **Nr of cell treated pts with long-term FUP** | **Functional method of FUP** | **Time between AMI and treatment** | **FUP EF** | **Sign.** | **FUP Infarct size** | **Sign.** | **Adverse events** |
| --- | --- | --- | --- | --- | --- | --- | --- | --- | --- | --- | --- | --- |
| **BOOST [13]** | BM-MNC | 5 | ic | 60 | 28 | MRI | 4.8±1.3 days | 55±2 vs 55±2% | n.s. | n.a. |  | 2 deaths of each group |
| **ASTAMI [14]** | BM-MNC | 3 | ic | 100 | 50 | MRI | 4-8 days | 47.5% vs 46.8% | n.s. | decrease of - 4.8% | n.s. |  |
| **MAGIC Cell [15]** | G-CSF-mobilized PBSCs | 2 | ic | 154 | 75 | MRI | n.a. |  | n.s. |  |  |  |
| **Cao et al. [16]** | BM-MNC | 4 | ic | 86 | 41 | Echo | 7 days | 50.5±8% vs. 46.4±8 | <0.001 | 26.3±0.7% vs 28.1±0.8% | n.s. | 3 (6.6%) MACCE events |
| **REPAIR-AMI [17]** | BM-MNC | 2 | ic | 204 | 100 | MRI (subgroup) | 3-7 days | 50.1% vs 43.6% | <0.01 | n.a. |  | 22% vs 33% |
| **TOPCARE-AMI [18]** | BM-MNC + circulating progenitors | 5 | ic | 59 | 55 | MRI (subgroup) | 3-7 days | from baseline 46±10% to 57±10% (5y) | <0.001 between baseline and 5y FUP | LE from 39.5±31.2 ml to 13.2±9.6 ml | <0.001 | 27% TVR, 1 death, 1 AMI |
| **Plewka et al. [19]** | BM-MNC | 2 | ic | 60 | 40 | Echo | 7 days | increase in 10% vs 4.7% | n.a. | n.a. |  | 23% vs 55% in BM-MNC and control groups |
| **MAGIC-3 [20]** | G-CSF-mobilized PBSCs | 5 | ic | 163 | 79 | MRI at 2 years FUP | n.a. | increase in 2.9±9.2% vs 1.7±9.9% | n.s. | n.a. |  | MACE significantly reduced in cell infusion group |
| **Mocetti et al. [21]** | BM-MNC | 5 | ic | 32 | 21 | Echo | 3 days | median 49% (11) vs 45% (8) | =0.015 | n.a. |  | 2 and 1 deaths in BMNC/control groups |

**Table B. Segmental contraction velocity determined by cardiac magnetic resonance imaging (n=26)**

|  | **Before BM-MNC therapy** | **1 year post BM-MNC therapy** | **5 years follow-up** |
| --- | --- | --- | --- |
| Basal anterior [cm/s] | 21.0±5.3 | 22.3±1.2 | 22.4±1.8 |
| Basal anteroseptal [cm/s] | 21.6±5.2 | 22.9±2.0 | 22.9±1.8 |
| Basal inferoseptal [cm/s] | 21.2±5.0 | 22.4±1.3 | 22.8±2.0 |
| Basal inferior [cm/s] | 21.4±5.4 | 22.7±1.6 | 22.9±1.9 |
| Basal inferolateral [cm/s] | 21.±5.27 | 22.9±1.9 | 22.9±1.8 |
| Basal anterolateral [cm/s] | 20.9±5.0 | 22.0±1.7 | 22.5±1.9 |
| Mid anterior [cm/s] | 18.0±4.7 | 19.2±1.9 | 19.2±2.2 |
| Mid anteroseptal [cm/s] | 18.5±4.9 | 18.7±2.1 | 19.1±1.9 |
| Mid inferoseptal [cm/s] | 19.1±4.6 | 20.4±1.0 | 20.5±2.0 |
| Mid inferior [cm/s] | 19.9±5.0 | 21.0±1.7 | 21.3±1.5 |
| Mid inferolateral [cm/s] | 19.2±4.5 | 20.4±1.2 | 20.1±2.0 |
| Mid anterolateral [cm/s] | 19.0±4.7 | 20.1±2.1 | 20.8±2.1 |
| Apical anterior [cm/s] | 15.5±4.0 | 16.3±1.8 | 18.1±1.5^a^ |
| Apical septal [cm/s] | 16.8±4.3 | 17.6±2.2 | 19.3±2.2 ^a^ |
| Apical inferior [cm/s] | 17.0±4.2 | 17.8±1.9 | 18.5±1.9 |
| Apical lateral [cm/s] | 16.6±4.2 | 17.4±2.1 | 17.9±2.1 |
| Apex [cm/s] | 12.1±3.4 | 12.9±2.2 | 14.8±1.4 ^a^ |

^a^ p<0.05 between baseline (before BM-MNC therapy) and 5-year follow-up values
